# EQUIFAT: A novel scoring system for the semi-quantitative evaluation of regional adipose tissues in *Equidae*

**DOI:** 10.1371/journal.pone.0173753

**Published:** 2017-03-15

**Authors:** Philippa K. Morrison, Patricia A. Harris, Charlotte A. Maltin, Dai Grove-White, Caroline McG. Argo

**Affiliations:** 1 University of Surrey, School of Veterinary Medicine, Faculty of Health and Medical Sciences, Guilford, United Kingdom; 2 Equine Studies Group, WALTHAM Centre for Pet Nutrition, Freeby Lane, Waltham-on-the-Wolds, Melton Mowbray, Leicestershire, United Kingdom; 3 University of Liverpool, Department of Obesity and Endocrinology, Faculty of Health and Life Sciences, Leahurst Campus, Chester High Road, Neston, Wirral, United Kingdom; 4 Biomics Ltd, Inverurie, Aberdeenshire, United Kingdom; Universidad de Zaragoza, SPAIN

## Abstract

Anatomically distinct adipose tissues represent variable risks to metabolic health in man and some other mammals. Quantitative-imaging of internal adipose depots is problematic in large animals and associations between regional adiposity and health are poorly understood. This study aimed to develop and test a semi-quantitative system (EQUIFAT) which could be applied to regional adipose tissues. Anatomically-defined, photographic images of adipose depots (omental, mesenteric, epicardial, rump) were collected from 38 animals immediately *post-mortem*. Images were ranked and depot-specific descriptors were developed (1 = no fat visible; 5 = excessive fat present). Nuchal-crest and ventro-abdominal-retroperitoneal adipose depot depths (cm) were transformed to categorical 5 point scores. The repeatability and reliability of EQUIFAT was independently tested by 24 observers. When half scores were permitted, inter-observer agreement was substantial (average κ_w_: mesenteric, 0.79; omental, 0.79; rump 0.61) or moderate (average κ_w;_ epicardial, 0.60). Intra-observer repeatability was tested by 8 observers on 2 occasions. Kappa analysis indicated perfect (omental and mesenteric) and substantial agreement (epicardial and rump) between attempts. A further 207 animals were evaluated *ante-mortem* (age, height, breed-type, gender, body condition score [BCS]) and again immediately *post-mortem* (EQUIFAT scores, carcass weight). Multivariable, random effect linear regression models were fitted (breed as random effect; BCS as outcome variable). Only height, carcass weight, omental and retroperitoneal EQUIFAT scores remained as explanatory variables in the final model. The EQUIFAT scores developed here demonstrate clear functional differences between regional adipose depots and future studies could be directed towards describing associations between adiposity and disease risk in surgical and *post-mortem* situations.

## Introduction

Adipose tissue is an active endocrine organ, secreting chemical messengers collectively termed adipokines into the circulation to mediate communication with other organs. White adipose tissue (WAT) is distributed in anatomically discrete depots throughout the body where it performs diverse functional roles. Specific depots range in function from those primarily providing structural support (e.g. the retrobulbar fat pad) and and thermal protection (e.g. subcutaneous WAT) to the more readily recognized role of WAT as a dynamic reserve of metabolic energy and water (for example mesenteric/omental WAT [1, 2]). The precise distribution of adipose tissues between depots within an individual, or ‘fat patterning’, has been related to disease risk in a number of domestic species and in man [3, 4]. For example, increased visceral (abdominal) WAT deposition measured by computed tomography (CT) has been clearly characterised as a risk factor for the development of cardiovascular and metabolic disease in man [5, 6].

Despite continued reports of a high prevalence of obesity in the UK population of leisure horses and ponies [7, 8], relatively little is known about functional differences between discrete adipose tissue depots in this species. Whilst the exact mechanisms remain unclear, obesity has been associated with an increased risk for the development of insulin dysregulation and the common systemic condition, laminitis, which initially presents as severe foot pain [9, 10]. Obesity can also have a negative impact on athletic performance and fertility [11, 12]. Understanding functional distinctions and differential health risks between the various adipose tissue depots requires a capability to evaluate these covert, internal WAT reserves. Body condition score (BCS) systems, originally intended as management tools for the assessment of flesh cover and subsequent meat yield in food animals, are now routinely applied to horses and ponies in the field to estimate ‘body fatness’. The various equine BCS systems which have been reported vary in terms of scale (0–5; [13] and 1–9; [14]) and descriptors. The system used by many researchers, including the current group [14], is a modification of the system first described by Henneke [11] and allows for the independent assessment of superficially palpable adipose depots in different body regions. When BCS data (using the system described in the current study) were compared to concurrently-collected data generated by the empirically validated deuterium oxide dilution method for a mixed breed population of horses and ponies [14], BCS proved to be a robust predictor of total body fat content up to BCS 6.8/9 [15]. Although BCS systems are useful for the assessment of ‘body fatness’ in *Equidae* especially when undertaken by experienced practitioners, they have clear limitations, including a degree of variation in BCS values recorded between different observers [16]. BCS systems, assess externally visible/palpable adipose tissues and cannot evaluate internal adiposity. Further, a BCS system used routinely by researchers failed to predict body fat content with any accuracy in obese animals (> BCS of 6.8 out of 9) [15]. Similarly, the ability to measure total body fat using the deuterium oxide dilution method is largely restricted to research settings and it cannot distinguish between body fat in specific anatomical regions. To quantify regional body fat distribution, powerful imaging modalities such as computed tomography (CT), dual-energy x-ray absorptiometry (DXA), and magnetic resonance imaging (MRI) have been widely applied in man [17, 18] and smaller food and companion species [19, 20]. The larger body size of horses has to date, prohibited the application of these methods for the quantification of regional adipose depots in living *Equidae*. Ultrasound-generated images have been used to determine the depth of adipose depots at specific anatomical sites. While these measures may provide useful indicators of regional adiposity, their application for the prediction of total body fat [21] has been questioned [22].

As a preliminary step towards improving our understanding of the different roles played by regional adipose depots in the horse, the present study aimed to develop and test a semi-quantitative *s*coring system for *post-mortem* evaluation of specific regional equine adipose depots. A second objective was to describe any associations between regional adiposity appraised *ante-mortem* via BCS, and *post-mortem* regional ‘fat scores’.

## Methods

Although animal procedures did not constitute an experiment as defined under the Animals (Scientific Procedures) Act 1986, all work was approved by the University of Liverpool’s Veterinary Research Ethics Committee. Three sequential studies were performed to address the objectives of 1) developing suitable descriptors to score 6 discrete adipose tissue depots (EQUIFAT scores) 2) testing the repeatability and reliability of these descriptors and to 3) using the system to initially evaluate any associations between these *post-mortem* ‘fat scores’ and *ante-mortem* BCS. Data were derived from animals presented at a commercial UK abattoir (LJ Potters, Taunton, Somerset) for reasons unrelated to this study (none of the animals were purpose-bred for meat production and to the best of our knowledge, none had been in hard work immediately prior to slaughter). Animals were slaughtered in accordance with EU legislations EC 852/2004, 853/2004 and 854/2004. All animals were in good general health and were deemed fit for slaughter after physical examination by the attending official veterinary surgeon (OVS).

### The development of EQUIFAT scores

**Fig 1:**
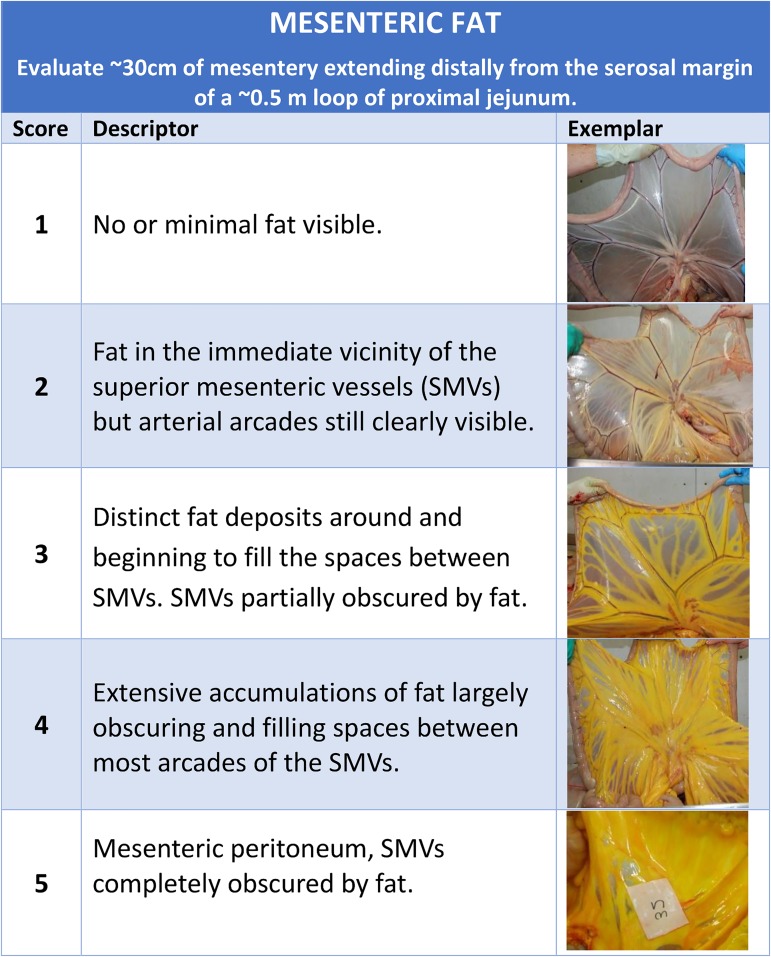
Example EQUIFAT scoring system for mesenteric adipose depot. The EQUIFAT scoring system was developed from the ranking of anatomically-defined depot-specific photographic images in order of increasing visually apparent adiposity. Detailed descriptors for each score (1–5) were generated and representative images are provided to aid in the use of the scoring system.

Anatomically defined photographic images of omental, mesenteric, epicardial and rump adipose depots were taken *post-mortem* from 38 animals between August and September 2012. The population comprised of mixed breed horses and ponies (26 horses 12 ponies; 23 mares 15 geldings) across the range of BCS (out of 9; [14]) as expected from a commercial abattoir setting (Mean BCS 5.0, SD 1.5, range 2.2–7.7) and was considered to be representative of the UK population of horses and ponies in terms of breed distribution, although Thoroughbreds were markedly over-represented in the abattoir setting (66% current study, 25% [23]. Photographs for each adipose depot were ranked in order of increasing ‘visually-apparent’ adiposity and a depot-specific 5 point scoring system, termed EQUIFAT (1 = least; 5 = greatest) was developed with detailed descriptors (Fig 1; S1 File). Representative images (*n* = 5) for each score were included with the descriptors for each adipose depot to facilitate the use of the scoring system. In addition to the above subjective scores, quantitative scores were also created for nuchal crest and abdominal retroperitoneal adipose depots. Depths (± 1mm) of the nuchal crest (the discrete adipose deposit extending from poll to withers, dorsal to the nuchal ligament and bounded laterally and dorsally by neck skin and subcutis) and abdominal retroperitoneal adipose depots were recorded at their cranio-caudal midpoints on the medial aspect of the left, split carcasses. The range of depths obtained for retroperitoneal and nuchal crest adipose depots were uniformly stratified and strata used as the basis of the ranges converted to categorical scores for these depots. The range of recorded depths were uniformly distributed and data were recoded as categorical scores (1–5) as follows: Crest: 1 = 0–2.99cm; 2 = 3–5.99cm; 3 = 6–8.99cm; 4 = 9–11.99cm; 5 = ≥12cm; Retroperitoneal: 1 = 0–1.99cm; 2 = 2–3.99cm; 3 = 4–5.99cm; 4 = 6–7.99cm; 5 = ≥8cm.

For assessment of BCS, six areas of the body (neck, withers, loin, tailhead, ribs and shoulder) are graded and each area is assigned a score from 1 (very poor) to 9 (extremely fat) based on detailed descriptors [14]. The average of the six values is calculated to provide a final, overall body condition score. All BCS measures were collected by a single, experienced observer.

### Testing the repeatability and reliability of EQUIFAT scores

The constraints of the commercial setting prohibited repeatability testing at the time of *post-mortem*. Therefore, the remaining 33 photographic images (excluding 5/38 presented with the descriptors) of each depot were randomised and used to create a slideshow for each depot. In order to assess the reliability and test the agreement between observers, the EQUIFAT scoring system was tested by a total of 24 individuals (17 veterinary surgeons, 5 clinical pathologists and 2 scientific researchers). Half of the respondents (randomly selected) were asked to use whole numbers only (1–5) and half were given the option of using whole or half scores. Each participant was informed of the nature of the study and provided with the images and the score descriptors. They were asked to assign a number between 1 and 5 (using half or whole numbers as above) for each image on a score sheet. Participants scored the images in isolation and were blinded to each other’s responses. To assess the repeatability of the scoring system, a random selection of four observers from each group (those using whole scores and those allowed to use half scores; 3 veterinary surgeons and 1 scientific researcher in each group) repeated the protocol at least two weeks after their first attempt (photographs were randomized to reorder from first attempt).

### Associations between EQUIFAT scores and BCS

Data for 207 animals were collected *ante-mortem* (BCS) and again immediately *post-mortem* (EQUIFAT) between August 2012 and January 2014 (Table 1). Information gathered *ante-mortem* included: age in years (passport), estimated withers height, breed-type, gender and BCS (out of 9) [8]. *Post-mortem*, carcass weight and EQUIFAT scores were recorded for omental, mesenteric, epicardial, rump, crest and retroperitoneal fats. Again, within the abattoir setting, all EQUIFAT scores were collected by a single experienced observer.

**Table 1:** Population of animals used in the current study as the test population used to develop the EQUIFAT scoring system (*n* = 38) and the population used to describe associations between EQUIFAT scores and BCS (*n* = 207).

	Test population (*n* = 38)	Whole population (*n* = 207)
	Average (Range)	
**BCS (/9)**	4.98 (2.2–7.7)	5.07 (2.3–8.3)
**Height**	151cm (102–178)	154cm (92–178)
	26 horses; 12 ponies	148 horses; 59 ponies
**Age**	10.1 years (3–20)	11.4 years (2–26)
**Gender**	15 Geldings, 23 Mares	70 Geldings, 137 Mares

### Data analysis

Statistical analyses were performed using STATA 12 (StataCorp, Texas). Statistical significance was set at p <0.05.

#### Intra-observer repeatability

For the four observers who repeated the assessment in each group, the number and percentage of exact agreement, along with score differences between the observers first and second attempts was calculated. The non-parametric Wilcoxon signed-rank test was applied to test the agreement between attempts with a predicted total difference between attempts of zero (100% agreement) for each observer. Pairwise kappa analysis using quadratic weights was then used to determine the agreement between observations. Quadratic weights assign less weight to agreement when comparative scores are further apart. Interpretation of kappa values is as follows: 0 = poor; 0.01 to 0.20 = slight; 0.21 to 0.40 = fair; 0.41 to 0.60 = moderate; 0.61 to 0.80 = substantial; 0.81 to 1.00 = almost perfect [24].

#### Inter-observer agreement

Kappa analyses were used to measure the agreement between observers for the 4 subjectively-scored adipose depots, beyond that expected by chance alone. Weighted kappa, using quadratic weights, was calculated for scores from each individual observer against those submitted by each of the other observers. The mean of these 11 weighted kappa values was recorded as the inter-observer agreement for that individual. This was repeated for each of the 4 adipose depots (omental, mesenteric, epicardial and rump fats).

Unweighted kappa was applied to each EQUIFAT score for each adipose depot in order to assess the repeatability each individual score.

#### Associations between EQUIFAT scores and BCS

Firstly, paired Student t-test was employed to assess any differences in age, BCS and height between the test population (n = 38) and the population in which they were nested (n = 207). Normality was assessed by visual appraisal of quintile-normal plots by transformation of each variable assessed. In order to describe associations between fat scores and BCS, two multivariable random effects linear regression models were fitted with BCS as the outcome variable and breed considered as a random effect. Early models, which included EQUIFAT crest and rump scores as explanatory variables demonstrated strong associations (data not shown) between these parameters and BCS. On consideration, this outcome could have been predicted as elements of the crest and rump EQUIFAT evaluations contribute one third of the overall BCS score. Therefore, to usefully describe associations between internal adiposity and BCS, crest and rump EQUIFAT scores were removed from further analyses. Models were fitted using a backward elimination strategy whereby a full model was built and then each variable removed in turn, a likelihood ratio test performed and the resultant P-value noted. The variable with the highest P-value was then omitted and the process repeated. This process was repeated until only variables with P < 0.2 remained in the model. The omitted variables were then added back in turn, starting with the lowest P-value, a likelihood ratio test performed after each addition, and the variable retained if P < 0.2. This process was continued until no further variables could be added, to produce the final model.

In Model 1, all physical attributes and remaining EQUIFAT scores (omental, mesenteric, epicardial and retroperitoneal) were offered to the initial model as explanatory variables. Model 2 was fitted using only the EQUIFAT scores in order to assess the association between internal fat depots and BCS irrespective of other physical characteristics. For both models, the intra-class correlation for the random effect variable (breed) was calculated as a measure of the variance attributable to the random effect.

Predicted marginal means were calculated from regression models and displayed graphically where appropriate.

## Results

### Test population

**Fig 2:**
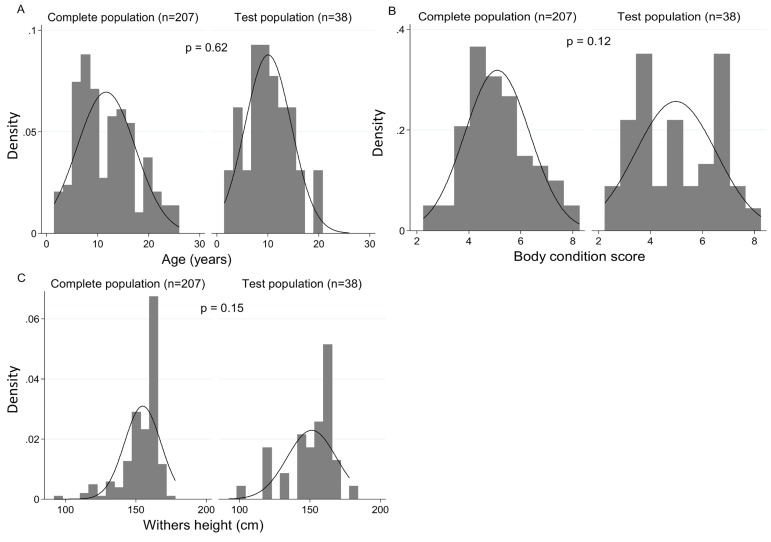
Population distributions of age, BCS and withers height in the test animals (*n* = 38) and the population in which they were nested (n = 207). Histograms were constructed with normal distribution line overlaid for age (A), body condition score (B) and withers height (C). Paired Students T-test was used to identify any differences between the populations.

A total of 207 animals presented at the abattoir were utilised in the present study. Results from thirty-eight animals were employed in development and validation of the EQUIFAT system. Paired Student t tests were employed to compare baseline characteristics (BCS, age, height) of these 38 to the population (*n* = 207) from which they were derived. The population of animals used to develop the EQUIFAT scoring system (*n* = 38) is described in Table 1. Fig 2 demonstrates there were no significant differences in attributes between the 38 test animals and the population (*n* = 207) in which they were nested.

### Intra-observer repeatability

Overall mean exact agreement between attempts for the four observer’s using half scores was similar for all four adipose depots, ranging from 10.8 (32.6%) for rump fat to 14.8 (44.7%) for epicardial fat out of the 33 images (Table 2). Mean exact agreement for the four observers using whole scores was greater than for those using half scores, with agreement ranging from 14.8 (44.7%) for epicardial fat to 20.8 (62.9%) for mesenteric fat (Table 3). In order to determine if any bias was present between observers attempts, each score they assigned for the second attempt was subtracted from the equivalent score from their first attempt. Generally, all eight observers who repeated using either whole of half scores scored higher in their second attempt for each depot, most notably for rump fat, with score differences of 0.49 and 0.34 respectively. For the other depots, mean difference between attempts was 0.15 (whole scorers) and -0.003 (half scorers) for mesenteric fat, 0.13 (whole scores) and -0.05 (half scorers) for omental fat, and 0.19 (whole scorers) and -0.10 (half scorers). Pairwise kappa analysis was very similar between the groups of observers using half scores (Table 2) and those using whole scores (Table 3). There was almost perfect agreement between scores for omental and mesenteric fats and substantial agreement for epicardial and rump fats.

**Table 2:** Intra-observer repeatability of the EQUIFAT scores for the four observers using half scores and assessing 33 images each of 4 adipose depots. A minimum of 14 days lapsed between attempts. Agreement data are presented for exact, 0.5 and 1 point differences between attempts. Kappa tests and p values for Wilcoxon sign-rank test are presented.

EQUIFAT	Observer ID	Exact agreement n/33 (%)	0.5 point difference n/33 (%)	1 point difference n/33 (%)	Mean observer difference in score between attempts	Pairwise kappa using quadratic weights	Wilcoxon signed-rank test of observer difference compared to zero p value
**Mesenteric**	1	13 (39.4)	19 (57.6)	1 (3.0)	-0.11	0.91	0.16
	2	13 (39.4)	12 (36.3)	8 (24.2)	0.24	0.81	0.02
	5	12 (36.4)	2 (6.1)	19 (57.5)	0.55	0.79	< 0.001
	11	12 (36.4)	10 (30.4)	11 (33.3)	-0.09	0.83	0.44
	**Overall Mean**	12.5 (37.9)	10.8 (32.6)	9.8 (29.5)	0.15	0.84	
**Omental**	1	13 (39.4)	15 (45.5)	5 (15.1)	0.11	0.91	0.10
	2	9 (27.3)	13 (39.4)	8 (24.2)	0.38	0.77	<0.001
	5	19 (57.6)	3 (9.1)	11 (33.3)	0.14	0.89	0.23
	11	14 (42.4)	9 (27.3)	9 (27.3)	-0.11	0.88	0.45
	**Overall Mean**	13.8 (41.7)	10 (30.3)	8.3 (25.2)	0.13	0.86	
**Epicardial**	1	10 (30.3)	15 (45.5)	7 (21.2)	0.29	0.74	0.01
	2	17 (51.5)	9 (27.3)	7 (21.2)	0.005	0.70	0.82
	5	16 (48.5)	1 (3.0)	12 (36.4)	0.23	0.69	0.30
	11	16 (48.5)	1 (3.0)	12 (36.4)	0.18	0.56	< 0.001
	**Overall Mean**	14.8 (44.7)	6.5 (19.7)	9.5 (28.8)	0.19	0.67	
**Rump**	1	10 (30.3)	16 (48.5)	5 (15.1)	0.05	0.76	0.90
	2	11 (33.33)	8 (24.2)	7 (27.3)	0.47	0.63	0.004
	5	9 (27.3)	1 (3.0)	19 (57.6)	0.74	0.54	< 0.001
	11	13 (39.4)	9 (27.3)	10 (30.3)	0.09	0.78	0.75
	**Overall Mean**	10.8 (32.6)	8.5 (25.8)	10.3 (31.2)	0.34	0.68	

**Table 3:** Intra-observer repeatability of the EQUIFAT scores for the four observers using half scores and assessing 33 images each of 4 adipose depots. A minimum of 14 days lapsed between attempts. Agreement data are presented for exact and 1 point differences between attempts. Kappa tests and p values for Wilcoxon sign-rank test are presented.

EQUIFAT	Observer ID	Exact agreement n/33 (%)	1 point difference n/33 (%)	Mean observer difference in score between attempts	Pairwise kappa using quadratic weights	Wilcoxon signed-rank test of observer difference compared to zero p value
**Mesenteric**	2	25 (75.8)	7 (21.2)	0.10	0.84	0.18
	8	16 (48.5)	17 (51.5)	-0.39	0.83	0.001
	9	23 (69.7)	10 (30.3)	0.18	0.90	0.06
	11	19 (57.6)	13 (39.4)	0.10	0.78	0.55
	**Overall Mean**	20.8 (62.9)	11.8 (35.6)	-0.003	0.84	
**Omental**	2	18 (54.5)	15 (45.5)	-0.33	0.85	0.004
	8	23 (69.7)	10 (30.3)	-0.06	0.90	0.53
	9	19 (57.6)	14 (42.4)	0.06	0.88	0.59
	11	25 (75.8)	8 (24.3)	0.12	0.92	0.16
	**Overall Mean**	18.8 (56.8)	8.3 (25.2)	-0.05	0.89	
**Epicardial**	2	12 (36.4)	20 (60.6)	0.42	0.41	0.004
	8	15 (45.5)	16 (48.5)	0.42	0.68	0.004
	9	14 (42.4)	14 (42.4)	0.36	0.64	0.04
	11	18 (54.5)	13 (39.4)	-0.27	0.72	0.06
	**Overall Mean**	14.8 (44.7)	9.5 (28.8)	-0.23	0.61	
**Rump**	2	19 (57.6)	11 (33.3)	0.45	0.72	0.001
	8	11 (33.3)	17 (51.5)	0.57	0.61	0.006
	9	11 (33.3)	18 (54.5)	0.79	0.58	< 0.001
	11	19 (57.6)	13 (39.4)	0.15	0.80	0.26
	**Overall Mean**	15 (45.5)	14.8 (44.7)	0.49	0.68	

### Inter-observer agreement

Weighted kappa analysis was employed to assess agreement within the two groups of 12 observers for each adipose depot (Table 4). For the four adipose depots, a weighted kappa value was generated for each observer against the 11 other observers and a mean weighted kappa was then recorded for each observer. As for the intra-observer agreement, the average kappa values obtained for each depot were very similar between those using half scores and those using whole scores (Table 4). For those using half scores, the overall mean weighted kappa was substantial for mesenteric (0.79; standard deviation (SD) 0.04), omental (0.79; SD 0.02) and rump (0.61; SD 0.07) fats, and moderate for epicardial fat (0.60; SD 0.07). For those using whole scores, the overall mean weighted kappa was substantial for mesenteric (0.79; SD 0.03) and omental fats (0.78; SD 0.04) and moderate for epicardial (0.54; SD 0.06) and rump fats (0.57; SD 0.08).

**Table 4:** Inter-observer agreement of the EQUIFAT scores.

Observer ID	Half scorers				Whole scorers			
	Mesenteric fat	Omental fat	Epicardial fat	Rump fat	Mesenteric fat	Omental fat	Epicardial fat	Rump fat
	Mean κ_w_ for each observer against 11 other observers				Mean κ_w_ for each observer against 11 other observers			
**1**	0.8	0.82	0.69	0.67	0.82	0.78	0.61	0.65
**2**	0.74	0.76	0.56	0.62	0.78	0.79	0.52	0.54
**3**	0.81	0.81	0.62	0.59	0.74	0.79	0.54	0.64
**4**	0.82	0.77	0.56	0.45	0.79	0.78	0.51	0.53
**5**	0.78	0.79	0.67	0.68	0.79	0.72	0.40	0.53
**6**	0.73	0.81	0.58	0.54	0.82	0.80	0.62	0.63
**7**	0.79	0.81	0.67	0.70	0.81	0.82	0.56	0.59
**8**	0.75	0.76	0.64	0.54	0.82	0.78	0.54	0.55
**9**	0.77	0.79	0.65	0.62	0.80	0.82	0.57	0.39
**10**	0.85	0.78	0.59	0.56	0.75	0.67	0.56	0.68
**11**	0.82	0.82	0.62	0.57	0.72	0.80	0.51	0.53
**12**	0.81	0.78	0.44	0.66	0.80	0.76	0.59	0.58
**Overall Mean (SD)**	0.79 (0.04)	0.79 (0.02)	0.61 (0.07)	0.60 (0.07)	0.79 (0.03)	0.78 (0.04)	0.54 (0.06)	0.57 (0.08)

A weighted kappa value was generated for each observer (n = 12) against each other individual observer. Mean weighted kappa are presented for each observer.

The application of un-weighted kappa analysis to describe the repeatability of the individual scores for each depot revealed substantial agreement between observers for a score of 5 for mesenteric fat (0.70) and a score of 1 for omental fat (0.61) for those using half scores (Table 5). The repeatability of the remaining scores was found to have either fair or slight agreement. For observers using whole scores there was moderate agreement for a score of 1 for mesenteric (0.41) and omental (0.49) fats and almost perfect agreement for a score of 5 for mesenteric fat (0.85), with moderate agreement for a score of 5 for omental (0.48) and epicardial fats (0.41) (Table 5). The repeatability of the remaining scores was found to have either fair or slight agreement. Lower agreement was observed for the scores between 1.5 and 4.5 for mesenteric and omental fats.

**Table 5:** Repeatability of individual EQUIFAT scores for each depot.

Score	Overall κ							
	Half scores				Whole scores			
	Mesenteric fat	Omental fat	Epicardial fat	Rump fat	Mesenteric fat	Omental fat	Epicardial fat	Rump fat
**1**	0.19 (Slight)	0.61 (Substantial)	0.06 (Slight)	0.20 (Slight)	0.41 (Moderate)	0.49 (Moderate)	0.16 (Slight)	0.17 (Slight)
**1.5**	0.10 (Slight)	0.09 (Slight)	0.07 (Slight)	0.18 (Slight)				
**2**	0.24 (Fair)	0.32 (Fair)	0.14 (Slight)	0.28 (Fair)	0.31 (Fair)	0.37 (Fair)	0.20 (Slight)	0.25 (Fair)
**2.5**	0.12 (Slight)	0.03 (Slight)	0.06 (Slight)	0.09 (Slight)				
**3**	0.12 (Slight)	0.21 (Fair)	0.10 (Slight)	0.10 (Slight)	0.24 (Fair)	0.29 (Fair)	0.13 (Slight)	0.17 (Slight)
**3.5**	0.15 (Slight)	0.10 (Slight)	0.04 (Slight)	0.03 (Slight)				
**4**	0.29 (Fair)	0.21 (Fair)	0.20 (Slight)	0.10 (Slight)	0.51 (Moderate)	0.32 (Fair)	0.21 (Fair)	0.15 (Slight)
**4.5**	0.25 (Fair)	0.10 (Slight)	0.08 (Slight)	0.02 (Slight)				
**5**	0.70 (Substantial)	0.40 (Fair)	0.35 (Fair)	0.36 (Fair)	0.85 (Almost perfect)	0.48 (Moderate)	0.41 (Moderate)	0.32 (Fair)

Repeatability of individual EQUIFAT scores for each depot. Un-weighted kappa analyses are presented.

### Associations between EQUIFAT scores and BCS

**Fig 3:**
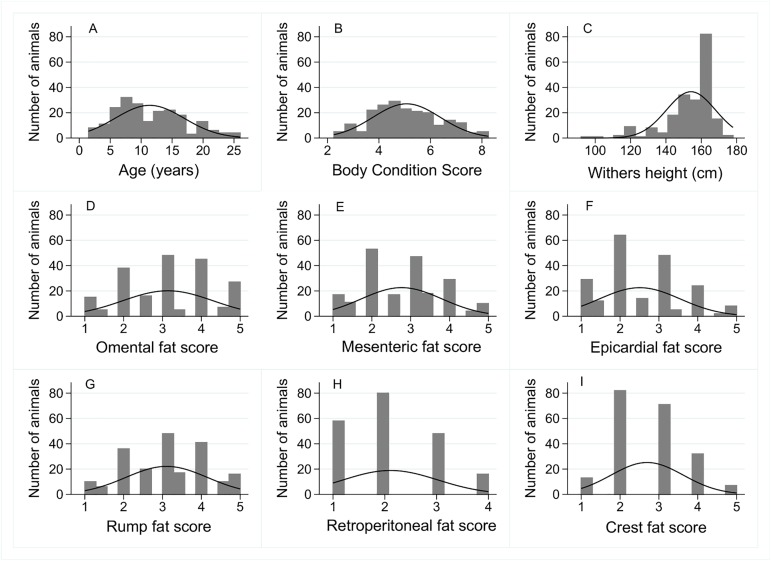
Distribution of physical attributes and EQUIFAT scores in the population of animals used to describe associations between EQUIFAT scores and BCS (*n* = 207). Histograms were constructed with normal distribution overlaid for age (A), BCS (B), withers height (C), omental fat score (D), mesenteric fat score (E), epicardial fat score (F), retroperitoneal fat score (G), rump fat score (H), and crest fat score (I).

The population of animals (*n* = 207) used for this part of the study are described in Table 1 and Fig 3. The animals were representative of the UK abattoir population in terms of gender, age, horse/pony split, and BCS. As outlined in the methods, both crest and rump fat scores were excluded from analysis as they were highly correlated with two components of the original BCS system, namely “neck” and “tailhead”.

**Fig 4:**
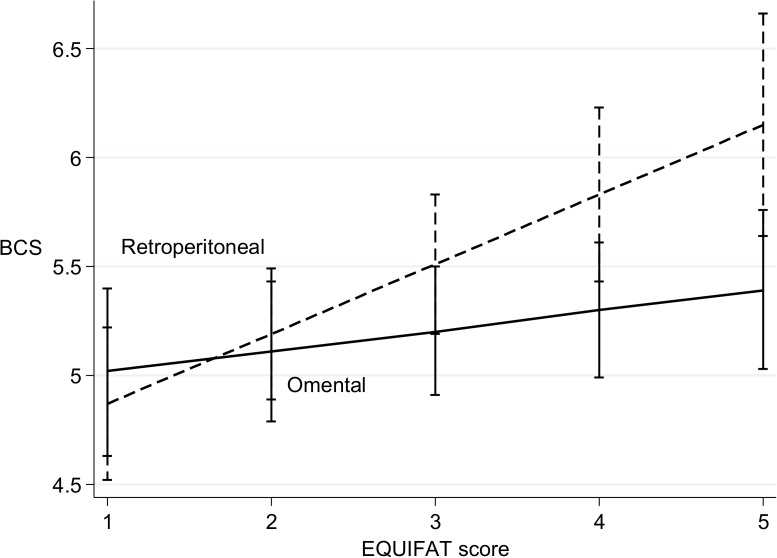
Marginal mean plots illustrating predicted changes in BCS with retroperitoneal and omental EQUIFAT scores. Marginal mean plots were created from the final multivariable model 1 to demonstrate predicted changes in BCS with increasing retroperitoneal and omental EQUIFAT scores. Error bars signify 95% confidence intervals.

Model 1 (Table 6) demonstrates there were strong positive associations between BCS and both carcass weight and retroperitoneal fat score. Withers height had a strong negative association with BCS. Age, gender, mesenteric and epicardial fat scores did not remain in the final model thereby demonstrating a lack of association with BCS. Model 2 (Table 6) was fitted to explore associations between the EQUIFAT scores and BCS. Variables remaining in the final model were retroperitoneal fat score and omental fat score, with neither mesenteric or epicardial fat scores remaining in the final model. In both models, the coefficient for retroperitoneal fat score was at least 3 times greater than that for omental fat. Fig 4 demonstrates the predicted marginal means generated from Model 1 and clearly indicate that for retroperitoneal depots, and to a lesser extent for omental fats, there was a trend for BCS to increase with each unit increase in specific fat scores.

**Table 6:** Associations between EQUIFAT scores and BCS.

Variable	Model 1 (Adj. R^2^ = 0.49) Breed attributable variance = 0.23 (95% CI = 0.07 to 0.54)			Model 2 (Adj. R^2^ = 0.24) Breed attributable variance = 0.31 (95% CI = 0.14 to 0.56)		
	Estimate β	95% CI	P value	Estimate β	95% CI	P value
Height (cm/10)	-0.62	-0.80 to -0.44	< 0.001			
Carcass weight (kg/10)	0.11	0.08 to 0.14	< 0.001			
Omental fat score	0.09	-0.02 to 0.21	0.10	0.16	0.03 to 0.28	0.02
Retroperitoneal fat score	0.32	0.17 to 0.47	< 0.001	0.48	0.32 to 0.64	< 0.001
Baseline	10.44	8.17 to 12.71	< 0.001	3.88	3.32 to 4.43	< 0.001

Two random effects, multivariable linear regression models were built with breed as a random effect. CI, confidence interval.

## Discussion

The current study firstly describes the development and testing of a novel fat scoring system for *Equidae*; the EQUIFAT scoring system, and secondly it demonstrates the application of the EQUIFAT scoring system to describe the relationship between internal adiposity and external body condition score. To date and to the authors’ knowledge, only one study has described associations between BCS and internal adiposity, whereby a strong, positive association was described between kidney, pelvic and heart fat with BCS in a group of horses and ponies presented for slaughter [25]. It was noteworthy that while obesity is prevalent among horses and ponies in the UK leisure sector [8], the population of animals presented for slaughter at a commercial abattoir in the current study was at variance with this. A greater proportion of animals assessed in the current study would be considered to be ‘normal’ or slightly underweight in terms of BCS than would be predicted had these animals been sourced from the numerically dominant leisure horse population. In order to test the repeatability and reliability of the EQUIFAT scoring system, kappa analysis was employed. Kappa analysis is a well-established and widely used method in numerous fields of scientific research and indicates the level of agreement either between or within observers beyond that expected by chance alone [26]. The results from the current study demonstrate almost perfect agreement in the repeatability of omental and mesenteric fat scores and substantial agreement for epicardial and rump fat scores, irrespective of whether half scores or whole scores were used. The data suggested that the EQUIFAT scoring system was robust when used on repeated occasions, although there did appear to be some bias between observers repeated attempts to classify the same images. On the whole, observers tended to score higher on their second attempt, although the average scoring difference remained below half a score in the majority of cases which was deemed as an acceptable difference by the authors. The inter-observer agreement between observers was also found to be substantial for mesenteric and omental fats for those using both whole scores and half scores; whilst the agreement was moderate to substantial for epicardial and rump fats. The reasons for the lower reliability of epicardial and rump compared to omental and mesenteric EQUIFAT scores are unclear but it may be that the degree of adiposity was clearer to distinguish in the photographs for these depots

The two groups of observers in the current study were instructed either to use whole scores only or were given the option of using half scores. There were no obvious differences in agreement between the two groups and from the feedback; it appeared that the EQUIFAT scoring system was applied with more ease when the use of half scores was permitted. Therefore, it would be recommended that half scores are allowed for future use.

The second part of this study applied the EQUIFAT scoring system to a large group of animals in order to describe associations between individual depot EQUIFAT scores and BCS. Due to the lack of availability of modern imaging modalities such as CT scanning for the quantification of internal fat in the live horse, using the EQUIFAT scoring system designed in the current study at *post-mortem* allowed the investigation of associations between external ‘body fatness’ (BCS) and internal fat deposition. In the current study, height was negatively associated with BCS, indicating that ponies had a greater BCS than horses, which agrees with other findings that pony breeds, especially UK native breeds of ponies are more at risk of obesity than Thoroughbred horses [8]. It is noteworthy that although regional differences in body fat distribution between men and women are well documented [27], there was no association between gender and BCS in the current study. This is likely due to the fact that the second gender in the current study was castrated males, although a recent study found no difference in BCS between a group of mares and stallions [28].Furthermore, a large epidemiology-based study identified that whilst ponies are at greater risk of obesity compared to horses, gender was not associated with obesity risk in this population [8].

A study describing associations between BCS and the anatomical distribution of adipose tissue through carcass dissection of 7 Welsh Section A mares identified that WAT was evenly distributed between internal and external sites and the relative sizes (mean % of recovered empty body mass [total body less digesta]) of some of the adipose depots described in the current study from smallest to largest were as follows: epicardial (0.08%), omental (0.41%), nuchal crest (0.65%), mesenteric (2.09%) and retroperitoneal (2.87%).

Taken together with the finding in the current study that retroperitoneal fat had a strong positive association with BCS suggests that this intra-abdominal depot may function as a long-term storage depot. Studies on retroperitoneal WAT function in the horse are limited, although ultrasound measurements of retroperitoneal fat depths were found to be associated with percentage body fat in a group of 77 horses and ponies [15]. However, there appears to be some debate in the literature regarding whether or not retroperitoneal adipose tissues should be classed as a ‘visceral fat’. In terms of venous drainage there are clear differences between peritoneal (omental and mesenteric) and retroperitoneal adipose tissues which could signify functional differences. Venous blood from peritoneal adipose tissues drains via the portal vein into the liver. Conversely, venous effluent from retroperitoneal adipose tissue depots drains into the renal circulation. Evidence from rodent studies supports the contention that retroperitoneal and peritoneal adipose tissues are physiologically distinct. For high-fat diet fed rats, exercise training decreased the response to isoproterenol-stimulated lipolysis in mesenteric but not retroperitoneal adipose tissues [29]. Depot differences have been also been demonstrated in the immune cell populations of the stromal vascular fraction of omental and retroperitoneal fats in mice [30]. A recent study in humans however, argues that retroperitoneal fat should be considered alongside omental and mesenteric fats to encompass the visceral depot as retroperitoneal fat was significantly correlated with metabolic syndrome and the number of metabolic abnormalities [31].

The visceral adipose depot (omental and mesenteric) is more metabolically and lipolytically active in humans and it has been shown that visceral fat is preferentially mobilised over subcutaneous fat during the initial stages of a very low calorie diet; although this depot bias is lost as weight-loss progresses [32]. Empirical data suggests that this may also be true for the horse. Circumferential body measures of ‘belly girth’ in a mixed-breed population of horses and ponies decreased during the course of a weight-loss trial, indicative of a loss of internal adiposity [33]. Furthermore, in the current study, omental fat score had a weaker association whilst mesenteric fat score had no association with BCS. These data suggest that, as for humans, these depots may function more as a short-term energy reserve. Human visceral fat incubated in primary culture secretes inflammatory cyctokines at a greater rate than subcutaneous fat [34]. For the horse, the nuchal crest may be an important source of inflammatory factors [35, 36]; although the relationship between circulating inflammatory factors and obesity is less clear in this species [37, 38].

A novel observation in the current study was the lack of any association between epicardial fat score and BCS. Epicardial fat is situated between the pericardium and myocardium and is thought to function to provide energy for the heart. Of note, epicardial fat was not associated with total extracted WAT from the carcass dissection of 7 Welsh mountain ponies across a range of BCS [39]. Importantly, epicardial fat has been shown to play a key role in the pathogenesis of coronary artery disease in humans [40] and an increased epicardial fat volume has been observed in patients with type 2 diabetes [41]. Additionally, mRNA for the brown fat marker, uncoupling protein 1 (UCP1) was expressed at higher levels in epicardial fat compared to other adipose depots [42], suggesting that this depot may have a further role in protecting the myocardium from hypothermia. Further studies may be warranted in the horse to determine whether this depot may have a role to play in insulin dysfunction or not.

The EQUIFAT scoring system was developed as an initial step towards wider applications to characterise fat patterning and clearly has broader applications in terms of furthering our understanding of regional adiposity and disease risk. The EQUIFAT system has the potential to capitalise on data readily collected during surgical interventions that require laparotomy. A relatively common cause of colic that requires laparotomy are strangulating lesions associated with the presence of a pedunculated lipoma arising from small intestine mesenteric WAT. A retrospective study conducted to assess the short-term survival rate of colic in a group of 300 horses and ponies that underwent exploratory laparotomy identified that 13% of those animals required surgical intervention due to intestinal strangulation by pedunculated lipoma, and the short-term survival rate of those 39 animals was 64.1% [43]. Interestingly, a recent study that evaluated associations between pituitary lesions, obesity and the presence of mesenteric lipomas in insulin-resistant horses found that whilst insulin-resistant horses had a higher frequency of mesenteric lipomas, there was no association between obesity and the frequency of mesenteric lipomas [44]. This finding combined with our finding in the current study that mesenteric fat scores were not associated with BCS would perhaps suggest that mesenteric fat scores as opposed to BCS may be associated with the frequency of mesenteric lipomas and may be an area for future study. Furthermore, breed is a known risk-factor for obesity in horses and ponies [8] and future studies could also be directed towards evaluating breed differences in the distribution of internal adiposity.

In summary, the current study outlines the development and testing of a novel depot-specific fat scoring system for horses and ponies ‘EQUIFAT’ which has been used to describe associations between regional fat depots and external BCS. The EQUIFAT scoring system proved to be robust when used on repeated occasions and on the whole there was very good agreement between observers when using the scoring system. Application of the scoring system on a large population of animals at *post-mortem* allowed associations to be made between BCS and the regional distribution of adipose tissue which demonstrated strong positive associations between BCS and retroperitoneal fat score, whilst there was no associations for mesenteric or epicardial fat scores. These associations indicate clear functional differences between the various adipose depots in terms of energy storage. Forward application of the EQUIFAT system would allow data collected at laparotomy or *post-mortem* to be collated with clinical findings. In combination, these methods could direct future studies towards furthering the understanding of the role played by regional adipose depots in obesity-associated pathologies such as laminitis, insulin dysregulation and pedunculated lipoma.

## Supporting information

S1 File EQUIFAT scoring system descriptors. (PDF)
