# Supplementary material for: EQUIFAT: A novel scoring system for the semi-quantitative evaluation of regional adipose tissues in Equidae
Source: PLoS One. 2017 Mar 15;12(3):e0173753. doi: 10.1371/journal.pone.0173753 (PMC5351866; doi:10.1371/journal.pone.0173753)
Supplement: S1 File — (PDF) [file pone.0173753.s001.pdf]

EQUIFAT: Regional adipose tissue scoring system

OMENTAL FAT

Evaluate ~30cm of omentum extending distally from the midpoint of the greater curvature of the stomach

Score	Descriptor	Exemplar
**1**	No or minimal fat visible	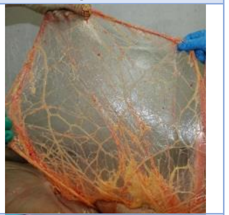
**2**	Fat in immediate vicinity of the gastroepiploic vessels (GEVs) but vessels still clearly visible.	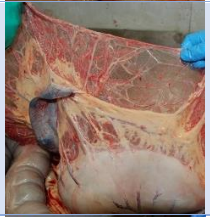
**3**	Distinct fat deposits around and beginning to fill the spaces between GEVs.<br>GEVs partially obscured by fat.	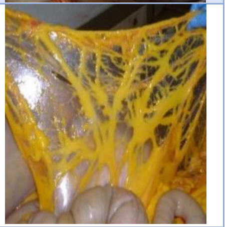
**4**	Extensive accumulations of fat largely obscuring and filling the spaces between most GEVs.	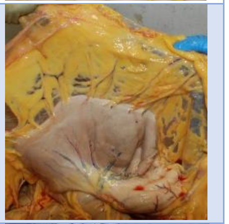
**5**	Omental peritoneum and GEVs completely obscured by fat.	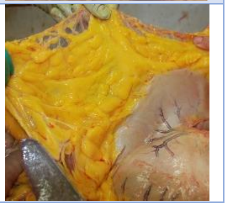

MESENTERIC FAT

Evaluate ~30cm of mesentery extending distally from the serosal margin of a ~0.5 m loop of proximal jejunum.

Score	Descriptor	Exemplar
**1**	No or minimal fat visible.	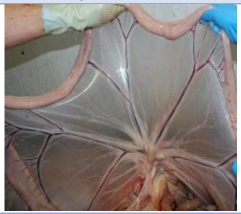
**2**	Fat in the immediate vicinity of the superior mesenteric vessels (SMVs) but arterial arcades still clearly visible.	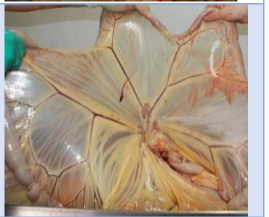
**3**	Distinct fat deposits around and beginning to fill the spaces between SMVs. SMVs partially obscured by fat.	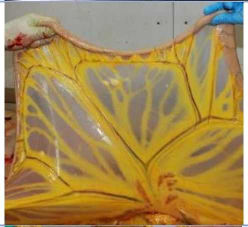
**4**	Extensive accumulations of fat largely obscuring and filling spaces between most arcades of the SMVs.	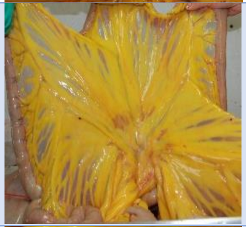
**5**	Mesenteric peritoneum, SMVs completely obscured by fat.	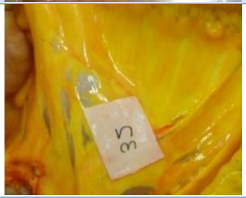

EPICARDIAL FAT

Evaluate left heart

Score	Descriptor	Exemplar
**1**	No or minimal fat visible	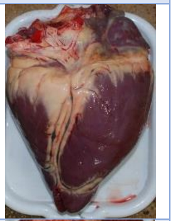
**2**	Fat limited to immediate vicinity of coronary groove (CG) and paracornial interventricular branch of left coronary artery (PIBLCA). Entire PIBLCA visible. Fat 'level' with adjacent myocardium.	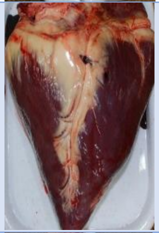
**3**	Fat 'tendrils' extend from vicinity of PIBLCA across myocardium. Proximal limits of PIBLCA partially obscured by fat. Fat minimally protruding over myocardial surface.	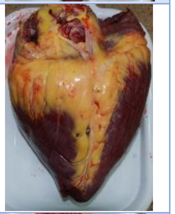
**4**	Lobular fat emanating from CG & PIBLCA only distal PIBLCA visible.<br>Fat protruding above myocardium but ≥50% of ventricular myocardium visible.	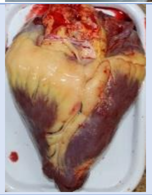
**5**	CG & PIBLCA completely obscured by lobular Fat in folded bulges with < 50% ventricular myocardium visible.	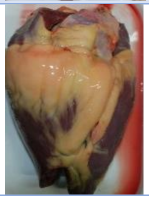

RUMP FAT

Evaluate dorsal rump from point of tailhead over loin

Score	Descriptor	Exemplar
**1**	No / minimal fat cover.<br>Flesh clearly visible.<br>(**<10% fat cover**)	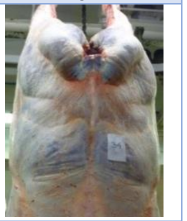
**2**	Visible fat on tailhead, flesh remains visible lower down rump<br>(**10 – 25% fat cover**).	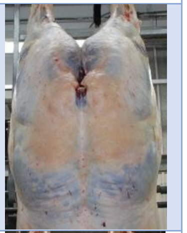
**3**	Small patches of flesh may remain visible.<br>(**25 – 50% fat cover**).	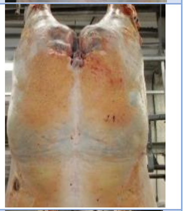
**4**	Flesh not clearly visible, fat may have bulging appearance.<br>(**50 – 75% fat cover**).	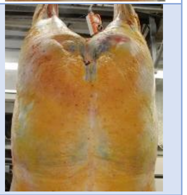
**5**	Fat appears thick and more protruding.<br>(**>75% fat cover**)	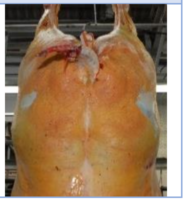

NUCHAL CREST FAT: Depth at craniocaudal midpoint

**Score**	**1**	**2**	**3**	**4**	**5**
**Depth (cm)**	**0 – 2.9**	**3 – 5.9**	**6 – 8.9**	**9 – 11.9**	**≥12**

ABDOMINAL RETROPERITONEAL FAT: Cr/Cau midpoint

**Score**	**1**	**2**	**3**	**4**	**5**
**Depth (cm)**	**0 – 1.9**	**2 – 3.9**	**4 – 5.9**	**6 – 7.9**	**≥8**
